# Muscle Synergy Analysis of a Hand-Grasp Dataset: A Limited Subset of Motor Modules May Underlie a Large Variety of Grasps

**DOI:** 10.3389/fnbot.2018.00057

**Published:** 2018-09-25

**Authors:** Alessandro Scano, Andrea Chiavenna, Lorenzo Molinari Tosatti, Henning Müller, Manfredo Atzori

**Affiliations:** ^1^Institute of Intelligent Industrial Technologies and Systems for Advanced Manufacturing (STIIMA), Italian National Research Council (CNR), Milan, Italy; ^2^Information Systems Institute, University of Applied Sciences Western Switzerland (HES-SO), Sierre, Switzerland

**Keywords:** muscle synergies, centroids, synergies clustering, hand grasps, spatial synergies, temporal components, NinaPro Database

## Abstract

**Background:** Kinematic and muscle patterns underlying hand grasps have been widely investigated in the literature. However, the identification of a reduced set of motor modules, generalizing across subjects and grasps, may be valuable for increasing the knowledge of hand motor control, and provide methods to be exploited in prosthesis control and hand rehabilitation.

**Methods:** Motor muscle synergies were extracted from a publicly available database including 28 subjects, executing 20 hand grasps selected for daily-life activities. The spatial synergies and temporal components were analyzed with a clustering algorithm to characterize the patterns underlying hand-grasps.

**Results:** Motor synergies were successfully extracted on all 28 subjects. Clustering orders ranging from 2 to 50 were tested. A subset of ten clusters, each one represented by a spatial motor module, approximates the original dataset with a mean maximum error of 5% on reconstructed modules; however, each spatial synergy might be employed with different timing and recruited at different grasp stages. Two temporal activation patterns are often recognized, corresponding to the grasp/hold phase, and to the pre-shaping and release phase.

**Conclusions:** This paper presents one of the biggest analysis of muscle synergies of hand grasps currently available. The results of 28 subjects performing 20 different grasps suggest that a limited number of time dependent motor modules (shared among subjects), correctly elicited by a control activation signal, may underlie the execution of a large variety of hand grasps. However, spatial synergies are not strongly related to specific motor functions but may be recruited at different stages, depending on subject and grasp. This result can lead to applications in rehabilitation and assistive robotics.

## Introduction

The use of the hands is one of the most crucial capabilities for daily activities. The loss of a hand can substantially reduce the quality of life of a person, since it strongly affects physical capabilities in performing activities of daily living (ADL) and it represents a relevant social problem considering that people with a major upper limb loss were ~41,000 in USA in 2005. The number of amputees is expected to double by 2050 (Atkins et al., 1996; Ziegler-Graham et al., 2008).

Hand grasps are mainly composed of two main stages: the reach-to-object and the grasp itself. The first phase is divided into two sub-phases, consisting of the transport of the hand done by the arm, whose motion law is characterized by a bell-shaped velocity (Fan et al., 2006), and the hand pre-shaping, required for adapting the hand to the object to grasp, which occurs after ~60–70% of the reaching phase (Hu et al., 2005). The grasp phase is determined by several parameters, including the force closure (force needed to close the hand around the object and to achieve a stable grasp), grasp stability (the ability to resist external forces), and grasp security (resistance to slippery objects, which is depending on the configuration of the grasp; Cutkosky, 1989; Cipriani et al., 2008). A third phase is reported in some articles (Liarokapis et al., 2013) and represent the release of the object; a fourth phase can be considered too, involving the return of the arm and hand to the rest position.

Hand grasps have been investigated mainly in the domain of finger joint kinematics and past studies have developed qualitative taxonomies to describe and cluster different types of grasps (Cutkosky, 1989). The main distinction among grasps was between power grasps and precision grasps but many other features can be taken into account for grasp characterization, such as the limb configuration for the task execution or the geometry of the object to grasp.

Considering the complexity of hand control, involving a remarkable number of degrees of freedom and redundancy, both at the muscle and skeletal levels, many studies in the literature applied feature extraction methods to identify a subset of the original data for an accurate description of hand functioning, even if reduced in dimensionality.

A recent study (Jarrassé et al., 2014) investigated a set of hand grasps by considering a 15-degree-of-freedom (dof) Cyberglove. The study used a Principal Component Analysis (PCA)-based technique for the extraction of kinematic motor synergies and showed that no more than 4 PCs are needed to explain ~95% of the total variation. The first and second PCs accounted for about 90% of data variation, leading the author to suggest that these two components might be enough to control (or even mechanically design) an upper-limb prosthesis, even if pattern refinement can be achieved by adding further PCs. In Patel et al. (2017), kinematic synergies were extracted by using a PCA-based algorithm. While the first PC accounts for more than half of the total variation, the rest is distributed across many PCs, indicating that a quite large set of motor modules is needed to reconstruct the original kinematics. Seven synergies were extracted in Thakur et al. (2008) for the explanation of >90% of the total variance of a set of hand-grasps and hand motions. A comprehensive study on hand grasps by Santello et al. (1998) suggests that the modules that underlie the control of the hand are basically two. However, the study also remarks that the remaining variation, accounted for by further synergies, is not due to noise but to motor control modules needed for fine tuning.

The fact that a limited number of modules may account for a large variety of grasps is thus commonly deduced from the literature. A recent study by Prevete et al. (2018) investigated the hypothesis of sparsity applied to kinematic synergies during hand grasps. According to this study, sparsity might be found both at the spatial synergy level (indicating that spatial modules may incorporate only some joints or muscles) and in the coordination of the synergies, in which only a reduced number of overlapping modules contribute to the execution of an action. A combination of the two conditions, called *double sparsity hypothesis*, can happen as well. This concept fits well with previous research on dimensionality reduction, with the addition that sparsity could partially explain the different number of synergies extracted in different studies (together with varying study designs).

Despite the kinematic patterns being exploited more often for hand analysis, some studies have investigated the dimensionality reduction problem from the point of view of muscle synergies. The muscle synergy approach is based on decomposition algorithms that identify groups of co-activating muscles (synergies) that are coordinated by time-varying activation commands. The extracted patterns may be influenced by several factors regarding sEMG, including fatigue, sweating, changes in electrode or arm positioning (Farina et al., 2014), clinical parameters of the subjects (e.g., level of the amputation, phantom limb sensation intensity; Atzori et al., 2016), the BMI (Atzori et al., 2014b), other anatomical characteristics of the subjects (Farina et al., 2002) or training in using myoelectric prostheses (Cipriani et al., 2011). Few studies addressed these effects until now, and the effect on the resulting muscle synergies. Considering upper limb synergies, Ortega et al. observed that synergy structure was conserved with fatigue, but interestingly synergy activation coefficients decreased on average by 24.5% with fatigue development (Ortega-Auriol et al., 2018). In Tagliabue et al. (2015) two-digit grasping is analyzed. A reduced number of modules (2–3) is needed to explain the largest part of the variation for each grasp and the correlation between muscle and kinematic primitives is suggested, justifying synergy-based analysis in both domains. Considering two arrays of sEMG-electrodes, positioned distally and proximally on the forearm, Castellini and van der Smagt (2013) found that the combination of 3 muscle synergies could account for a set of 5 hand grasps, on both sets of the electrodes. The “main synergy” represents a “global, indistinct” co-activation pattern, while the other two synergies account for dorsal and ventral patterns, respectively.

Overduin et al. (2008) used the time-varying muscle synergy model to analyze a set of 25 grasps of two monkeys and found that three synergies could explain 71% of the total sEMG variation for proximal muscles, 83% for the wrist and extrinsic hand muscles and 81% among intrinsic muscles. The first of the three synergies was linked to the muscles involved in the reach phase operated by proximal muscles and distal flexors, the second was characterized by bimodal activation of distal muscles and the third, more related to the transport of the object, featured by proximal muscles and distal extensors.

The main challenge of using muscle synergies to analyze hand grasps is represented by the impossibility to track all the muscles involved in the grasps, as hand muscles are hard to acquire due to their small size, which can easily produce cross-talk, and due to encumbrance of probes/wires on the palm of the hand that can prevent a physiological grasp execution. Nevertheless, the reduction of the dimensionality is still a crucial process for the comprehension of the patterns underlying hand use and grasps. In fact, motor modules are considered to be the basis of motor control organization at the neural level (Schmidt, 1975; d'Avella et al., 2006). Furthermore, once recognized, the basic modules might be employed as references for the study of motor control, to evaluate pathological conditions and to control prosthetic devices. Dexterous, naturally controlled surface electromyography (sSEMG) prostheses would better allow amputees to perform personal needs such as eating or using tools. Prosthetics companies and scientific research are advancing toward this, but dexterous naturally controlled prosthetic hands are not yet available, in the market as well as in scientific research mainly due to control problems (Atzori and Muller, 2015) related to robustness. Clinical parameters of the amputation were demonstrated to affect control capabilities (Atzori et al., 2016). In order to foster the improvement of control systems for sEMG hand prostheses, a publicly available dataset for robotic hand prosthesis control (the Ninapro database^1^) was released in 2014 (Atzori et al., 2014a), and extended with several additional datasets afterwards (Krasoulis et al., 2017; Pizzolato et al., 2017). Currently, the database includes over 120 subjects (including 11 trans-radial amputees), repeating as naturally as possible up to 53 hand movements with several acquisition setups ranging in price from a few hundred to several thousand dollars. The aim of Ninapro is to foster the improvement of the field by allowing the development and test of advanced machine learning methods. However, the path to natural control of dexterous prosthetic hands can also be paved by the simplification of the problem, for instance via the identification of a set of motor primitives sufficient to control a comprehensive set of hand grasps.

The application of muscle and postural hand synergies to myoelectric hand prostheses development and low level control was recently suggested in literature and tested in specific settings, while high level control strategies are still not extensively explored. The application of postural hand synergies to hand prostheses development is particularly evident in the development of the PISA/IIT Softhand, a robotic hand actuated by a single motor (Catalano et al., 2014). The application of postural hand synergies to low level control approaches can be defined as controlling a dexterous robotic hand with few (usually 4) independent input signals that modulate some of the first synergies (usually the first one-two) in the robotic hand, leading the robotic hand to reproduce several hand grasps (Matrone et al., 2010, 2012; Segil and Weir, 2013).

In the literature, there are several open points regarding hand grasp synergies that can be investigated in more detail. Some of the more refined studies, providing state-of-the-art methods, involve a large variety of grasps but a limited number of subjects, or map a reduced number of grasps compared to the ones that are needed for daily life activities, lacking generalization of results. Furthermore, a limited number of studies focuses on muscle patterns rather than on hand kinematics. Lastly, most studies focused especially on the spatial organization of motor modules, while the temporal components were less analyzed.

Following the previous considerations, the aim of this study is threefold. First, to provide a set of benchmark muscle hand synergies extracted from the publicly available NinaPro database, that includes a considerable number of subjects while repeating a comprehensive number of hand grasps; second, to evaluate the effects of the reduction of dimensionality of the dataset on the accurateness in reconstructing the original dataset of synergies; third, to characterize the spatial and temporal features of the subjects included in the dataset.

## Materials and methods

### Acquisition set-up

**Figure 1:**
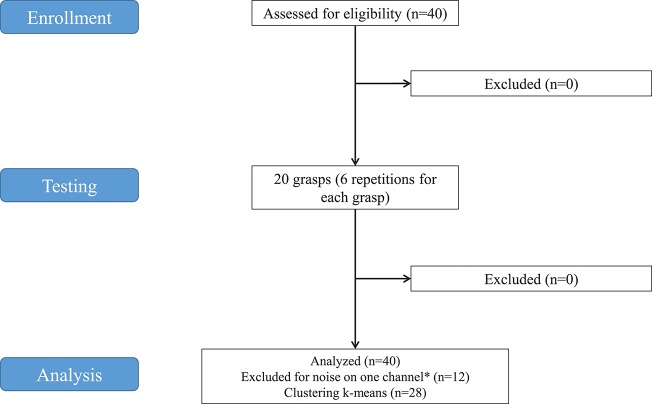
Study flowchart. *Twelve subjects were excluded from analysis because noise was found on at least one of the SEMG channels in some grasps. The decomposition algorithm applied to extract synergies would be influenced, even in case of removal of the affected channels from the analysis. Consequently, 12 subjects were discarded.

**Figure 2:**
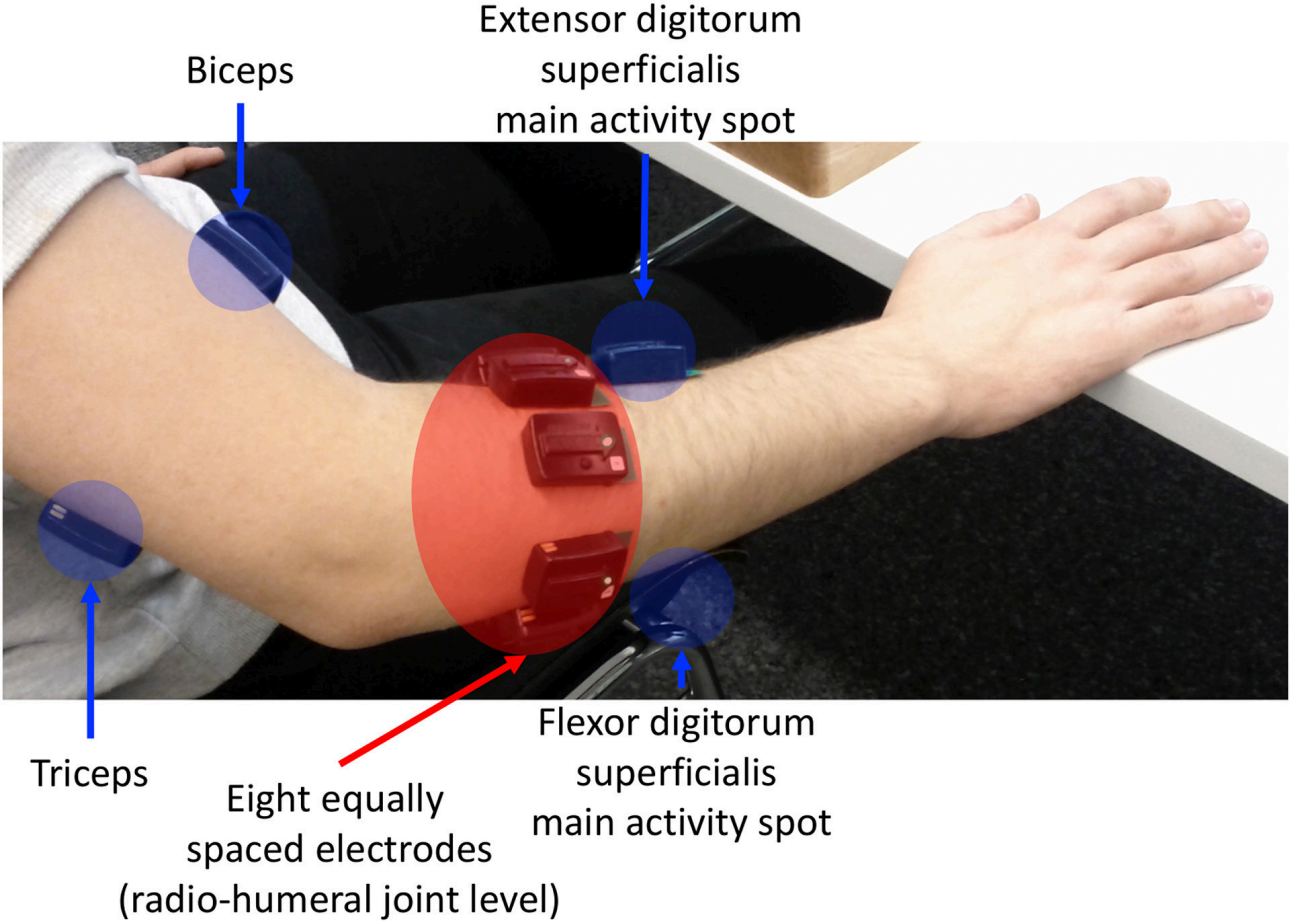
sEMG electrode placement: an array of 8 equally spaced electrodes was worn at the forearm level (labeled f1-f8), two probes on finger flexors and extensors, and on biceps caput longus and triceps caput medialis, according to the protocol introduced in the Ninapro database (Atzori et al., 2014a).

The flow-chart of the study is portrayaed in (Figure 1). The acquisition setup included 12 surface EMG (sEMG) electrodes and a data glove. The sSEMG electrodes were a double differential Delsys Trigno wireless system, measuring the myoelectric signals at 2 kHz with a baseline noise inferior to 750 nV RMS. The Trigno integrated a 3-axes accelerometer sampled at 148 Hz. Electrode positioning was performed with the aim of combining precise anatomical positioning (DeLuca, 1997) and a dense sampling approach (e.g., Fukuda et al., 2003). Eight electrodes were equally spaced around the forearm at the height of the radio-humeral joint. Four electrodes were placed on the main activity spots, respectively, of the flexor and the extensor digitorum superficialis, the biceps and the triceps brachii, which were identified by palpation by trained researchers by trained researchers (Figure 2). The data glove (CyberGlove II, CyberGlove Systems LLC 2) allowed to measure hand kinematics using 22-sensors. Considering that the primary objective of this study was to characterize the hand grasps rather than the dynamics of the reaching phase at proximal level, the choice of the NinaPro database is reasonable, since it includes recordings from extrinsic hand muscles.

### Participants

The data used in this experiment were from the publicly available NinaPro database that currently includes 7 datasets of sEMG and kinematic data from over 120 subjects (including 11 trans-radial amputees), performing (or imagining to perform) up to 53 different hand movement (Atzori et al., 2016). The datasets used for this study were from the second dataset (DB2), which includes 40 intact subjects. A 28-subject subset of the original dataset was used for this study. The subjects include 19 males, 9 females; 24 right handed, 4 left handed; average age 29.64 with standard deviation 3.1 years (data summarized in Table 1). Twelve subjects were excluded from the analysis because the proper extraction of synergies was prevented by noise of the sEMG channels. The decomposition algorithm applied to extract synergies would have been influenced, even in case of removal of the affected channels from the analysis.

**Table 1:** Summary of the demographic data of the involved subjects.

**Subjects**	**Gender**	**Age**	**Laterality**
**DEMOGRAPHIC DATA**			
*N* = 28	19M-9F	29.64 ± 3.1	24R-4L

### Experimental protocol for acquisition

**Figure 3:**
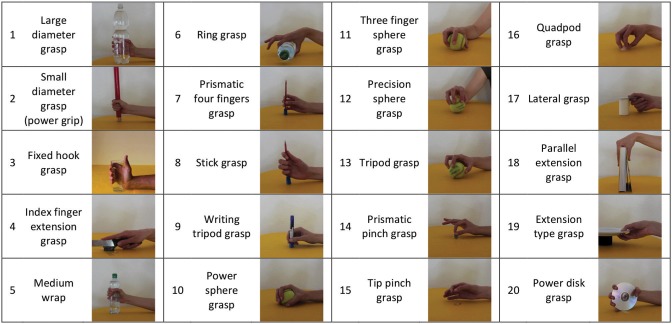
The 20 grasps considered in this study are shown. They provide a comprehensive mapping of the repertoire of hand grasps available to human subjects, and are stored in the publicly available Ninapro Database.

This section briefly describes the acquisition protocol. For more details about the protocol, please refer to Atzori et al. (2014a). During the experiment, the subjects were asked to sit at a desktop with the arms relaxed on the table and to repeat a set of movements with their right hand as naturally as possible. The entire experiment included 49 movements plus rest, divided into three exercises and extracted from the ADL literature, thus including movements from categories, such as personal needs, eating or use of tools (Smurr et al., 2008). In this work, we consider only the set of hand grasps, i.e., the first 20 movements of the second exercise (Figure 3). The subjects were asked to repeat the movements represented in short films that were shown on the screen of a laptop with their right hand and they were asked to concentrate on mimicking the movements rather than on exerting high forces. Each movement was repeated 6 times, with each repetition lasting 5 s and separated by the other movements by 3 s of rest. The experiment was approved by the Ethics Commission of the Canton Valais (Switzerland) and before data acquisition, the subjects were given a thorough written and oral explanation of the experiment itself and were asked to sign an informed consent.

### Data analysis: synergies extraction

The Data Analysis was fully performed with Matlab 2014a with custom-developed software. First, kinematic recordings (“restimulus” signal of the NinaPro database) were used to separate movement phases. Data from 12 sEMG channels were bidirectionally high-pass filtered at 50 Hz (Butterworth filter, 7th order) to remove motion artifacts, rectified, Hilbert-transformed (Matlab hilbert), low-pass filtered with a cut-off frequency of 10 Hz (Butterworth filter, 7th order) to remove noise with mono-directional filtering. sEMG data from each subject and each trial were pooled in single aggregated matrices and synergies were extracted using the non-negative matrix factorization (NMF) algorithm (Cheung et al., 2005; Tresch et al., 2006). The NMF decomposes the sEMG matrix into the product of two matrices, the first one representing time-invariant, neurally coded synergies (*w*_*i*_), and the second one representing time-variant activation commands for each synergy (*c*_*i*_), as in Equation (1):

(1) $$\begin{array}{l} EMG\left(t\right)=\sum_{i=1}^{N}c_{i}w_{i} \end{array}$$

where, for each of the recorded muscles, sEMG(t) represents the sEMG data at time *t* and *N* is the total number of extracted synergies.

The order of the factorization *r* was chosen, increasing from 1 to 50 (to limit the dimensionality for synthesis). For each *r*, the NMF algorithm was applied 1,000 times in order to avoid local minima. The repetition accounting for the highest variance of the signal was chosen as the representative of order *r*. The number of synergies was chosen as the minimum r explaining at least 90% of the variance of the signal (Clark et al., 2010). Further synergies were added only if the total amount of variation was increased of at least 5% for each further synergy.

### Synergy clustering

In the literature of motor synergies, standard analysis methods may include the definition of clusters to group synergies according to their spatial composition. The set of extracted synergies can be clustered to obtain a limited number of spatial patterns, each one represented by a centroid (mean spatial synergy).

In this work, the extracted synergies were included into a single cluster analysis. Grouping all the modules could lead to complex matching between each spatial component and the corresponding motor function (Scano et al., 2017). In fact, it was reported in Roh et al. (2013) that synergies related to the same motor function may split into two or more clusters. As a consequence, the correspondence between the phases of the grasps and the motor synergy recruitment is not always clearly identifiable. In fact, in the majority of the cases, the synergy prevailing in terms of magnitude of the temporal components is the one characterizing the moment of the grasp hold. However, a relevant number of subjects may show patterns more complex to identify.

However, performing the clustering procedure on the whole dataset allowed to provide a comprehensive overview of all the modules involved in hand grasping tasks. Furthermore, a comprehensive mapping of hand grasps is proposed by considering the whole dataset for analysis.

The cluster analysis was conducted using the k-means clustering algorithm. The algorithm was applied to an aggregated matrix containing the whole dataset of muscle synergies extracted from all subjects. Each clustering order, ranging from 1 (minimum) to 50 (maximum), was tested by repeating the algorithm 200 times and selecting the best solution for each order according to the metrics described in the following section.

### Selection of the number of clusters

The selection of the appropriate number of clusters (mean spatial synergies, each one represented by a cluster centroid) was made by pondering the following metrics (Bora et al., 2014):

1. The Mean Euclidean Distance (MED) of the population from the reference centroids, indicating the quality of the clustering, as a synthetic index for each clustering order. The lower the Mean Euclidean Distance, the better elements fit into their cluster.
2. When the k-means clustering procedure is applied, the number of desired clusters N must be specified. Defining as M the number of elements to be clustered (in this case, the number of spatial synergies), N can range between 1 and the total number of the clustered elements (1<=N<=M).
3. When *N* = 1, the clustering procedure classifies a population within a single group: thus, the cluster solution 1 is (implicitly) the mean of a population, and corresponds to the lowest level of precision in approximating a population with a clustering procedure. Following the previous considerations, the Normalized Euclidean Distance (NED) was computed by considering the cluster solution 1 as the source of maximum clustering error, which was set to 1. Thus, the NED for each clustering order *i* was computed as:
  (2) $$\begin{array}{l} NED\left(i\right)=\frac{MED\left(i\right)}{MED\left(1\right)} \end{array}$$
4. The slope of the Normalized Euclidean Distance (NED') is NED derivative. NED indicates how the precision of the cluster analysis increases when increasing the order of the clustering.

Each of the previous three metrics can be considered for the choice of the clustering order, by imposing a threshold on the reconstruction accuracy.

Whatever metric is selected, the choice is driven by the principle of using a parsimonious number of clusters for synthesis power (the lowest possible number of clusters, given a reasonable descriptive precision). The threshold selected by the experimenters in this work was 5%. Consequently, the number of clusters was selected as the minimum number needed to have the NED < 0.05.

The hypothesis that justifies the use of cluster analysis is that the dataset can be represented with a chosen number of cluster centroids depending on the maximum error that the experimenter is willing to accept. Depending on the application, the tolerance can be increased or reduced, describing the original dataset of motor modules with a specific level of precision (and a choice of dimensionality).

### Spatial and temporal components analysis

The characterization of the obtained mean spatial synergies was furtherly specified by considering all the pairwise dot products between their compositions. Each temporal component, initially associated with its respective spatial synergy, was matched to its relative centroid after cluster identification. Then, all the temporal components were averaged to extract a mean temporal component for each cluster, representing a mean activation of the spatial synergy in time. Finally, the characterization of temporal components was concluded by considering the correlations between the mean temporal components.

### Summary of outcome measures and statistics

Given the aims of the study (see Introduction): “First, to provide a set of benchmark muscle hand synergies extracted from publicly available data^1^ including a considerable number of subjects that perform a comprehensive number of hand grasps; second, to evaluate the effects of the reduction of dimensionality of the dataset on the accurateness in reconstructing the original dataset of synergies; third, to characterize the spatial and temporal features of the sample of subjects included in the dataset,” the following outcome measures were defined:

*Outcome 1:* Definition of the complete dataset of extracted muscle synergies of healthy subjects in freely executed grasps; *methods and statistics:* NMF algorithm for factorization; 90% of the VAF + minimum slope 0.05 for each further extracted synergy.
*Outcome 2:* Definition of cluster centroids for muscle synergies in freely executed grasps; *methods and statistics:* k-means clustering; lowest normalized Euclidean distance to define the number of centroids.
*Outcome 3a:* Characterization of the spatial composition of the centroids; *methods and statistics:* dot products between pairwise centroids to assess their difference in composition.
*Outcome 3b:* Characterization of the temporal features of the centroids; *methods and statistics:* Pearson correlations between temporal components.

## Results

### Extracted synergies

**Figure 4:**
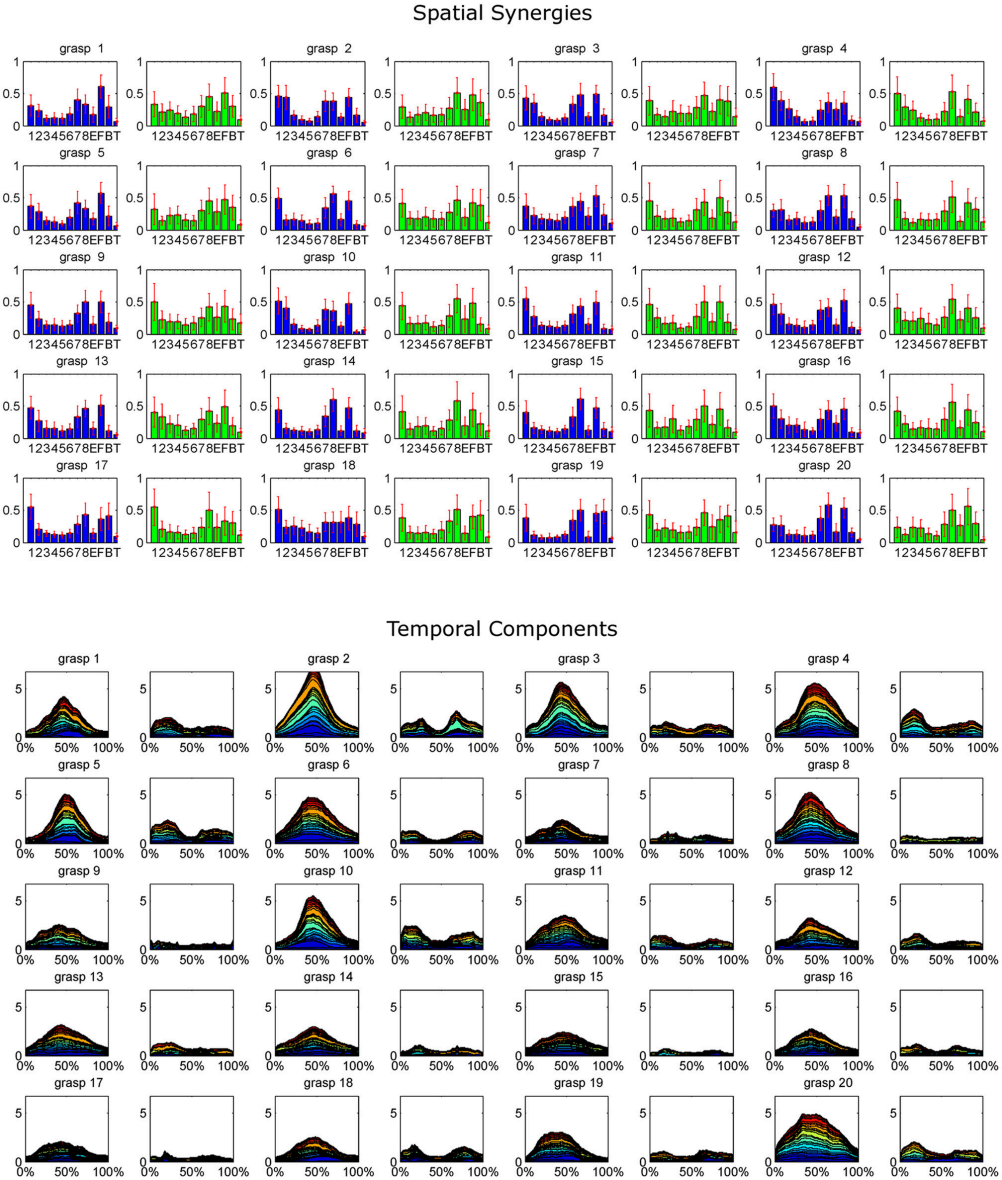
The whole dataset of synergies extracted for each grasp is synthetically reported, coupled with the corresponding cumulated temporal components. For each grasp (numbered 1–20 as in the order shown in Figure 2), the mean spatial synergies are reported. The mean spatial synergies are computed by averaging the spatial synergies grouped by matching each subject's spatial synergies according to the Pearson's Correlation coefficient computed on the temporal components. Only the first two modules are reported for each grasp (module 1, reported in blue, exploited during the grasp phase, and module 2, depicted in green, used mainly in the pre-shaping and release phases). Mean spatial synergies are also coupled with the cumulated mean temporal components that modulate in time the mean spatial synergies, plotted as percentage of the normalized duration of each movement.

The extracted synergy dataset is summarized in Figure 4 by portraying the mean spatial synergy compositions and cumulated temporal component profiles. Synergies were grouped within grasps, and matched according to the similarity of their temporal components, computed with the Pearson's correlation coefficient. For compactness of the representation, only the first two synergies of each extracted dataset were portrayed (while, three modules were extracted in some grasps).

### K-means cluster order selection

**Figure 5:**
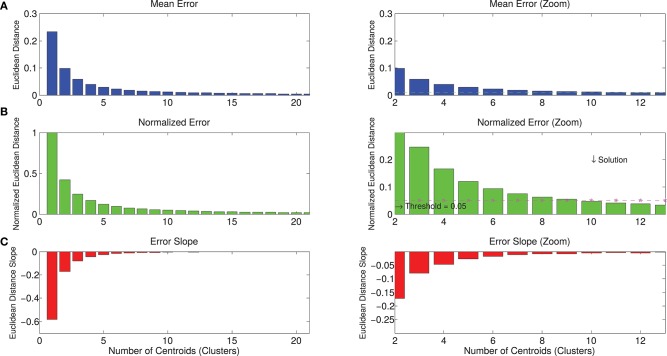
Metrics for the selection of the correct number of clusters for the description of the dataset. Panel **(A)** describes the mean Euclidean distance between the centroids identified with the k-means algorithm and the synergies that belong to that centroids. Increasing the number of clusters, the mean Euclidean error decreases. Panel **(B)** reports the normalized error, considering the solution of order 1 as the maximum approximation error (when the dataset of spatial synergies is approximated with its mean—SSm). Increasing the order of the clustering, the mean error is progressively reduced. Panel **(C)** shows the derivative of the error (slope), indicating the entity of the reduction of the error in relation to the increase of the number of clusters. Right panels show a zoomed view of left panels. They show that, by imposing a threshold of a maximum tolerable mean error, the solution corresponding to a lower number of clusters can be selected. In the present study, the maximum normalized Euclidean error was reasonably set at 0.05^*^SSm, corresponding to a 10-cluster solution.

The whole dataset of spatial synergies, which is composed of 966 extracted modules, was clustered according to the k-means algorithm, with a clustering order ranging from 1 to 50. Figure 5 shows the graphs with the metrics used for the choice of a reasonable number of clusters as synthetic representation of the spatial synergies of the dataset. Increasing the order of the clustering leads to a monotonic decrease of the NED. Thresholding the NED (at 0.05, as explained in the methods), only 10 clusters are needed to approximate the original dataset. It can also be observed that a further increase of the order of the clustering provides only slightly increased precision in describing the dataset.

### Clustering on spatial synergies and temporal components analysis

**Figure 6:**
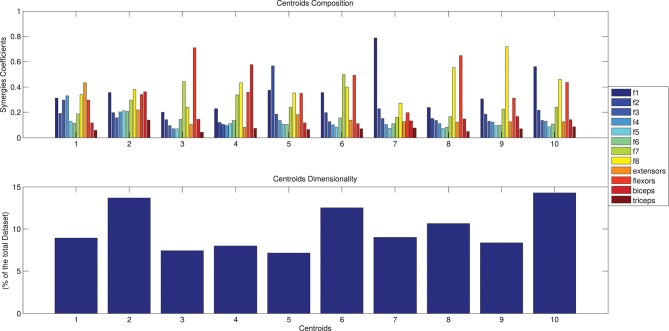
The identified centroids (mean spatial synergies). Hypothesizing a desired maximum error of 0.05 (normalized in respect to the solution of order 1), a solution of 10 clusters is found. Ten motor modules are thus enough to describe with good level of precision the original dataset. The 10 clusters composition are reported in **(A)**, along with the number of elements belonging to each cluster, expressed as percentage of the original dataset, in **(B)**.

**Figure 7:**
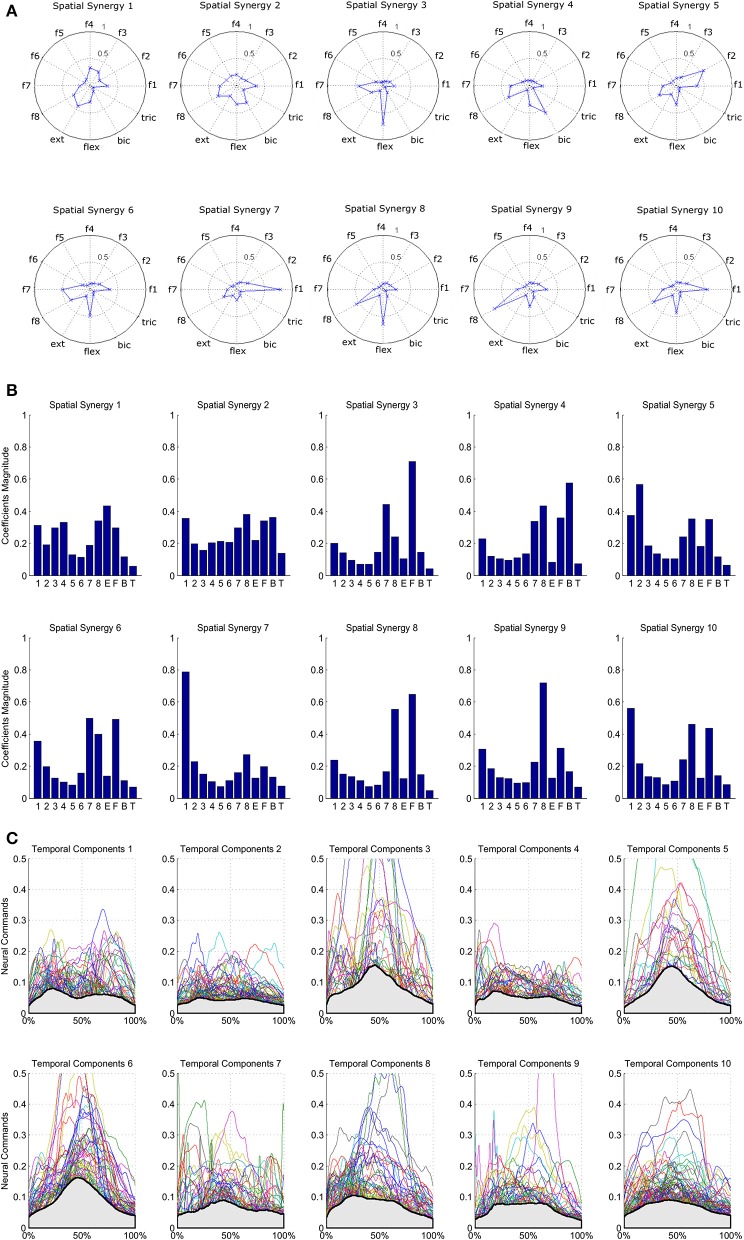
Summary of the extracted spatial synergies and mean temporal components associated with each spatial synergy. Spatial synergies are represented in a polar plot **(A)** and with histograms **(B)**. Temporal components are depicted in **(C)**, and mean temporal components are shown in light gray.

**Figure 8:**
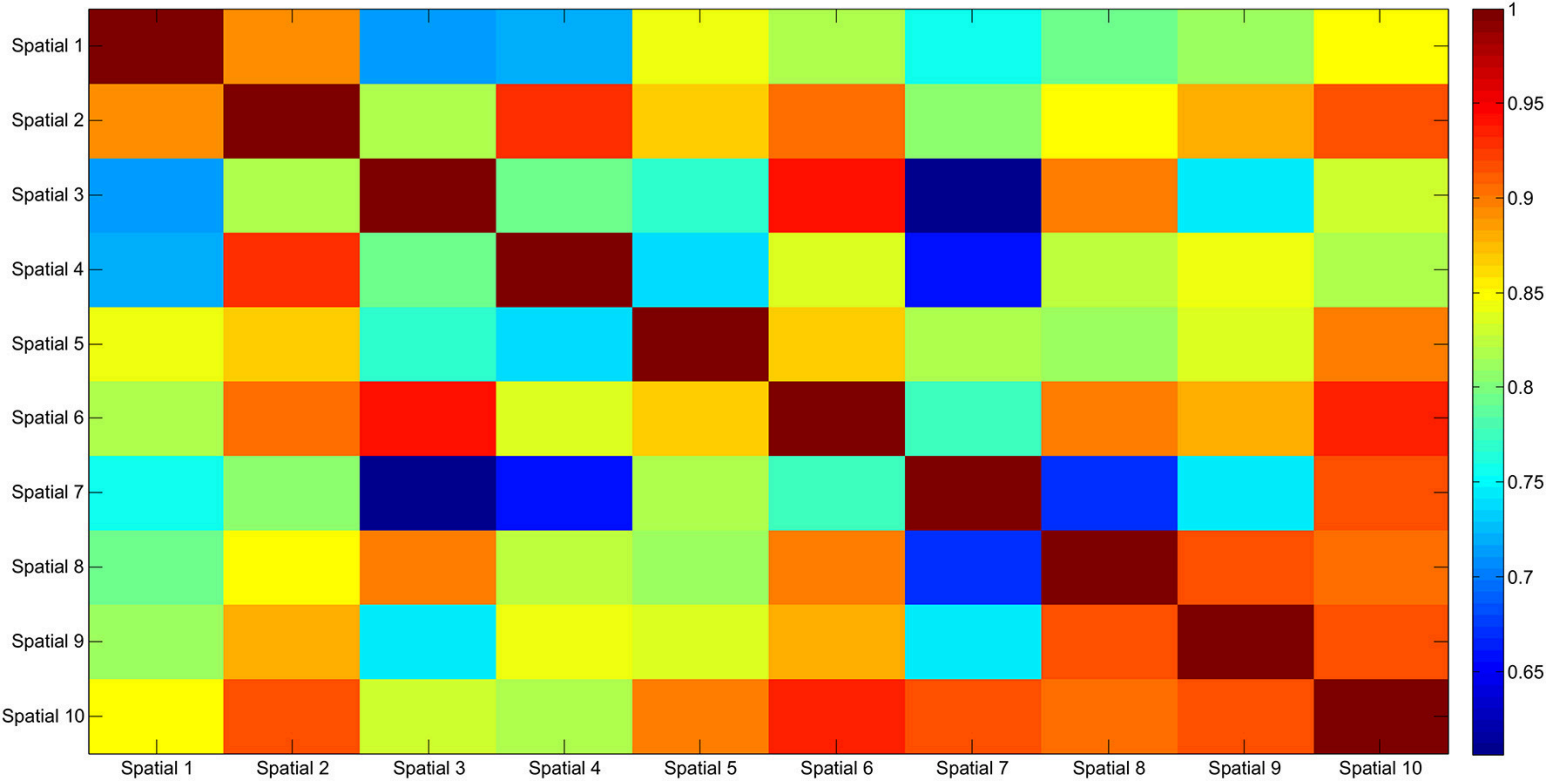
Mean spatial synergy correlation matrix. The matrix identifies the variability between all the pairs of spatial modules, assessed with the dot product. The paired similarity is always >0.60, indicating that the some muscle components are shared between centroid pairs.

**Figure 9:**
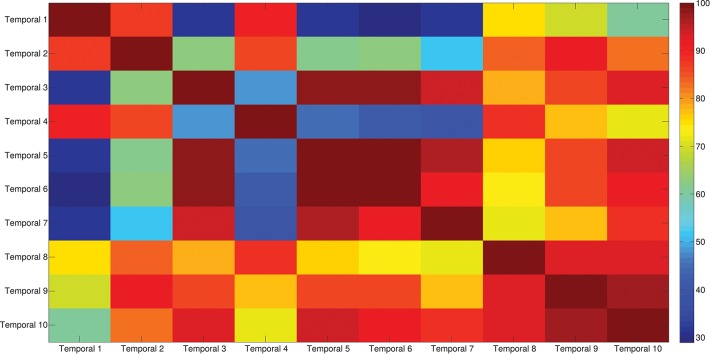
Temporal components correlation matrix. The matrix identifies the variability between all the pairs of temporal components associated to the mean spatial synergies, assessed with the Pearson's Correlation coefficient. The paired correlation ranges from 0.30 to 1, indicating that some modules are exploited with very similar (shared) control signals, while other modules are controlled with different timing.

The results of the clustering procedure are shown in Figure 6. The 10 identified centroids (mean spatial synergies) are portrayed (composition coefficients), along with the number of synergies of the original dataset that are addressed to each centroid, expressed as percentage of the original dataset. It can be seen that the extracted synergies are quite uniformly distributed on the centroids, each one representing between 7 and 15% of the original dataset of motor modules. Figure 7 depicts a polar and histogram-based representation of the extracted mean spatial synergies, along with the associated mean temporal components. The temporal components are shown for each of the spatial modules referring to each of the centroids, along with their mean. Analysis of temporal components shows that some centroids are found mostly in the central phase of the grasp (e.g., centroid 3 and centroid 6), while others mainly in the pre-shaping and release phases (e.g., centroid 1 and centroid 2). Following these results, in order to provide characterization of the summarized groups of motor modules, the similarity of mean spatial synergies and temporal components was assessed as well. Figure 8 shows the similarity, expressed as the dot product, among all the pairwise mean spatial synergies. It can be seen that the mean spatial centroids have a pairwise dot product ranging from 0.65 to 1, indicating that some muscle groups are shared between several patterns. Similarly, Figure 9 shows the correlation matrix between each temporal component, expressing the temporal relation that links each spatial synergy to the others. In this case, results show high variability, and indicate that some mean temporal components are very closely related to others (e.g., temporal components 5 and 6), while others are very different (e.g., temporal components 1 and 6). These results are critically analyzed in the following paragraphs.

## Discussion

### On the extracted synergies

An interesting result of this study is that, with the used method for synergy extraction, a number of modules ranging from 1 to 3 is sufficient for reconstructing the majority of the original sEMG in each grasp. As a consequence, a limited number of patterns is needed to achieve a grasp, which is a relevant result considering the availability of high redundancy at the muscle and kinematic level. This is seen in some patterns that are often repeated and especially in the co-activating group composed of f1-f8-finger flexors that are very often grouped together, especially in the hold phase. In most of the cases, two activation patterns are recognizable: a strong co-activation, often (but not always) corresponding to the grasp/hold phase, and two minor co-activating patterns in the pre-shaping and release phases that are often grouped in a single synergy. This result is particularly interesting considering that only two electrodes were not positioned on the forearm (respectively, biceps and triceps) and comparing the results with the results obtained by d'Avella et al. (2006) and Liarokapis et al. (2013). In these studies, the biceps is activated during the reaching phase in confirming that it is indeed an active reaching component, being active in the pre-shaping and release phase. This result suggests that the pre-shaping and release synergies may represent hand opening, before (pre-shaping) and after grasping. The number of phases seems to be in accordance with those proposed by Liarokapis et al. (2013) and seems to reproduce on the hand part of the results obtained in previous studies in terms of time varying muscular synergies for shoulder and arm. Furthermore, it should be remarked that the movements considered in this study were not performed against gravity, reducing consistently the involvement of shoulder muscles.

While not extensively discussed in this paper, the remarkable repeatability of the temporal components might be a further motor-control feature aimed at simplifying muscle coordination, as a strategy exploited by the CNS to perform hand grasps. These results are in accordance with the previous findings in the literature, that showed that, in respect to the original dimensionality of the control, the number of modules underlying grasps is probably strongly reduced (Santello et al., 1998; Overduin et al., 2008; Jarrassé et al., 2014).

### Cluster analysis and control of precision

On a comprehensive dataset of 20 grasp types, performed by 28 healthy subjects, 10 spatial motor modules, properly elicited in time, are enough to describe the whole dataset with good accuracy, generalizing through subjects. Such results are coherent with the notion that the central nervous system may embed a modular structure that relies on a limited number of predefined co-activation patterns to produce motor outcome at the hand level. These findings are in accordance with previous results that demonstrated that a small subset of synergies can generalize across tasks and suggest that they represent basic building blocks underlying natural human hand motions (Thakur et al., 2008).

The main spatial synergies were not directly linkable to specific grasp types or motor functions, suggesting that the spatial modules that can be employed for the execution of different grasp types. Furthermore, each spatial module can be elicited at different stages. Together with previous findings, these results suggest that grasp types and muscle synergies may not be univocally related: some muscle patterns may be used for different grasp types or, vice versa, the same grasp might be controlled with slightly different muscle synergies depending on the subject.

These results also reflect some intrinsic features of the human grasping related to proximal forearm and hand muscles control. This study suggests that a large variety of hand grasp types can be performed with a limited number of patterns. However, it should be considered that the proposed protocol was meant for applications related to control of prosthesis for trans-radial amputees, measuring the activity of proximal muscles. Coherently, previous studies sharing proximal muscle based protocols showed that a few basic patterns are responsible for a variety of grasp types (Castellini and van der Smagt, 2013). On the contrary, considering a fine recording of the muscles of the hand more differentiated patterns may be observed, even if due to the difficulty of recording muscle activity directly on the hand the motor primitives related to the hand are computed and analyzed in a kinematic domain (Jarrassé et al., 2014; Prevete et al., 2018).

### Temporal components

The analysis of temporal components underlines that spatial patterns may be recruited at different stages of a grasp, with variability related both to the subject who executes the grasp and the type of grasp. This result is confirmed by the high correlation of the temporal components of many clusters. However, mean temporal components suggest that some patterns are more often used during the grasp phase with a monophasic, bell-shaped activation profile, while other patterns are biphasic and usually activate when the hand opens, so in the approaching/pre-shaping phase and in the release phase rather than in the middle of the grasp. Such findings can be taken into account for several applications related to high level robotic hand and prosthesis control, as described in section Impact of the Muscle Synergy Dataset.

### Impact of the muscle synergy dataset

A limited number of motor modules (e.g., 10), properly elicited in time can approximate the entire dataset for all subjects with high accuracy (5% error in respect to approximating the dataset with its mean, in the case of 10 motor modules). Ideally, each movement considered in the experiment can potentially be reproduced as a combination of spatial synergies, thus providing prostheses with higher dexterity (a higher number of movements that can be controlled) starting from a set of a few robustly controlled modules. Hand muscle synergies may be applied to high level control approaches, consisting of training subjects to reproduce and modulate the sEMG patterns that correspond to the muscular hand synergies (or combinations of them) and apply pattern recognition algorithms to recognize the results. This strategy may be an alternative to the control systems currently described in literature. As said in the introduction, robotic hands that reproduce hand movements by modulating the main postural hand synergies have already been presented in literature (Matrone et al., 2010, 2012; Segil and Weir, 2013). However, high level control systems have not been extensively studied. Developing high level control systems based on time dependent muscle-hand synergies and training subjects to perform them may link the subjects' intentions with the movement of a robotic hand naturally, by exploiting the same synergies. Such result may lead to natural myoelectric control of robotic hands, a challenge currently not yet achieved in literature. If replicated on hand amputees, this result can potentially have applications in rehabilitation and assistive robotics in order to improve the control of dexterous prosthetic hands, by joining robotics and neuroscience findings (Santello et al., 2016).

Usually, in machine learning the training data (used to train a model) and the test data (used to test it) are taken from the same distribution. However, this is not always easy, in particular when using deep learning approaches that require large amounts of data for training. To overcome distribution mismatches, transfer learning and domain adaptation approaches have been used in several domains, including computer vision (Saenko et al., 2010; Tommasi et al., 2010), and natural language processing (Ben-David et al., 2010; Daumé et al., 2010). In myoelectric control, several studies explored the use of previous models from different subjects to reduce the amount of required training data (Farina et al., 2002; Tommasi et al., 2013; Patricia et al., 2014), but performance increase was not confirmed after proper model optimization (Gregori et al., 2017). The fact that the motor modules are common to the subjects can provide physiological foundations to include within the prosthesis a subject-independent motor memory. Prosthesis control could then be produced as “plug and play,” improve control robustness for a specific subject through successive calibration, and improve its adaptability to other subjects too. In this context, properly choosing the motor modules and the movements to be reproduced (in order to maximize dexterity, robustness and correspondence to ADLs) is potentially interesting to improve the rehabilitation capabilities of hand prostheses. However, it is an open question in the field of how exactly extracted synergies are mapped into motor functions: previous studies employing clustering procedures or synergy combination theories (Prevete et al., 2018) showed that the mapping between “physical space” of the end effector and the extracted muscle synergies may be due to different exploitation mechanisms.

In this study, it is proposed that a linear combination of centroids, properly activated by their temporal components, can be enough to reconstruct the physical space of the end effector in a large variety of grasp types with high accuracy. However, the authors are aware that the noticeable reduction of the original dataset implies that the original sEMGs are reconstructed with a pre-determined level of precision. The proper tradeoff between accuracy and synthesis needs to be tested in future work where the reduced dataset is integrated into a real control system.

Despite the potential provided by the muscle synergy analysis, several limitations and issues related to the method should be considered. Recent studies reported that pre-processing, including filtering and normalization techniques, might lead to different results and interpretation of the data (Shuman et al., 2017; Kieliba et al., 2018). While it is commonly accepted to normalize the duration of the tasks to a common phase axis, as it was done in this study, uniform guidelines for EMG pre-processing for synergies extraction are still missing in the literature. Consequently, pre-processing could be a source of data misinterpretation. Furthermore, the insurgence of fatigue was not inspected in this study, while it was demonstrated in the literature that fatigue may influence the recruitment of synergies, even if their spatial composition is preserved (Ortega-Auriol et al., 2018). As described in the section Introduction, several factors may have an effect on sEMG signal and make synergies tough to be generalized. Those may include fatigue, despite the acquisition protocol was carefully designed to induce low fatigue on subjects, even in the case of patients (Atzori et al., 2014a), and future developments should also consider these variables for a complete assessment.

Lastly, the model of human grasps described in this paper can potentially provide insights for calibrated interventions of rehabilitation robotics. Several implications can be found considering neurological or orthopedic rehabilitation of the hand (Bissolotti et al., 2016; Vanoglio et al., 2017). In recent studies, the exploitation of devices for hand rehabilitation has shown to lead to promising, therapeutic results that can be further enhanced by training muscle synergy-oriented exercises, based on a detailed knowledge of motor synergies (Scano et al., 2018).

## Conclusion

In this paper, muscle synergies were extracted from the recordings of a publicly available dataset. The extracted synergies were clustered from a cohort of 28 subjects executing a variety of hand grasps. The synergies are often characterized by two temporal activation patterns: a strong co-activation corresponding to the grasp/hold phase, and two minor co-activating patterns related to hand opening (visible in the pre-shaping and release phase). The conclusions of this article suggest that a limited number of time-dependent motor modules, correctly elicited by a control activation signal, may underlie the execution of a large variety of hand grasps. However, spatial synergies are not strongly related to a specific motor functions but have a sparse recruiting timing.

## Author contributions

AS designed the experiment, wrote the software for synergy extraction and clustering, elaborated the data, and wrote the paper. AC participated to data analysis and interpretation, and wrote the paper. LM participated to data analysis and wrote the paper. HM acquired the NinaPro database and wrote the paper. MA acquired the NinaPro database, participated in the design of the study and to data interpretation, and wrote the paper.

### Conflict of interest statement

The authors declare that the research was conducted in the absence of any commercial or financial relationships that could be construed as a potential conflict of interest.

## Footnotes

^1^Ninapro database: http://ninapro.hevs.ch
